# Investigating the roles and functions of Rab23 in primary ciliogenesis

**DOI:** 10.1186/2046-2530-1-S1-P33

**Published:** 2012-11-16

**Authors:** YS Lim, BL Tang

**Affiliations:** 1 National University of Singapore

## 

The small GTPase Rab23 has been shown to be an antagonist of the mouse developmental Sonic hedgehog (Shh) signalling pathway. Beyond embryogenesis, Rab23’s enrichment in adult rodent brains and dysregulated levels of Rab23 observed in various tumors, suggest other undefined postnatal functions. Since the primary cilium is crucial in regulation of the Shh signalling pathway, it is possible that Rab23 has a role in the genesis or functional aspect of the primary cilium. We checked Rab23’s localization and activity in association with the primary cilium. Evidently, only overexpressed Rab23 wild-type and its constitutively active form, Rab23Q68L, were significantly associated with the primary cilia. Both Rab23S23N (dominant negative) and Rab23T150A (a phosphorylation site mutant) were not localized to the cilia. With regards to ciliogenesis, the Rab23SN overexpressing population appeared to have a significant decrease in ciliated cells. Furthermore, Rab23 wild-type and Rab23QL overexpressing ciliated cells appeared to have a significant increase in cilial lengths. Similarly, cells overexpressing Evi5-L (ecotropic viral integration site 5-like), the only known GAP (GTPase activating protein) for Rab23 thus far, also had a significant reduction in the number of ciliated cells and a decrease in cilial lengths. We propose that Rab23’s localization to the primary cilium could be regulated by phosphorylation at the 150th threonine residue, and its ciliary localization could be involved in the regulation of cilial morphology and genesis.

